# Aminopeptidases in Cardiovascular and Renal Function. Role as Predictive Renal Injury Biomarkers

**DOI:** 10.3390/ijms21165615

**Published:** 2020-08-05

**Authors:** Félix Vargas, Rosemary Wangesteen, Isabel Rodríguez-Gómez, Joaquín García-Estañ

**Affiliations:** 1 Depto. Fisiologia, Fac. Medicina, Universidad de Granada, 18071 Granada, Spain; fvargas@ugr.es (F.V.); isabelrg@ugr.es (I.R.-G.); 2 Depto. Ciencias de la salud, Universidad de Jaén, 23071 Jaén, Spain; rwanges@ujaen.es; 3 Depto. Fisiologia, Fac. Medicina, IMIB, Universidad de Murcia, 30120 Murcia, Spain

**Keywords:** urinary aminopeptidases, biomarkers, arterial hypertension, renal function

## Abstract

Aminopeptidases (APs) are metalloenzymes that hydrolyze peptides and polypeptides by scission of the N-terminus amino acid and that also participate in the intracellular final digestion of proteins. APs play an important role in protein maturation, signal transduction, and cell-cycle control, among other processes. These enzymes are especially relevant in the control of cardiovascular and renal functions. APs participate in the regulation of the systemic and local renin–angiotensin system and also modulate the activity of neuropeptides, kinins, immunomodulatory peptides, and cytokines, even contributing to cholesterol uptake and angiogenesis. This review focuses on the role of four key APs, aspartyl-, alanyl-, glutamyl-, and leucyl-cystinyl-aminopeptidases, in the control of blood pressure (BP) and renal function and on their association with different cardiovascular and renal diseases. In this context, the effects of AP inhibitors are analyzed as therapeutic tools for BP control and renal diseases. Their role as urinary biomarkers of renal injury is also explored. The enzymatic activities of urinary APs, which act as hydrolyzing peptides on the luminal surface of the renal tubule, have emerged as early predictive renal injury biomarkers in both acute and chronic renal nephropathies, including those induced by nephrotoxic agents, obesity, hypertension, or diabetes. Hence, the analysis of urinary AP appears to be a promising diagnostic and prognostic approach to renal disease in both research and clinical settings.

## 1. Aminopeptidases in the Renin–Angiotensin System

The renin–angiotensin system (RAS) plays an essential role in blood pressure (BP) control, via vascular, renal, brain, and other mechanisms. Abnormalities in RAS activity may lead to the development of arterial hypertension and other cardiovascular and renal diseases. Blockade of this system is an effective therapeutic measure against numerous diseases, and RAS compounds have been found in the kidney, brain, and other tissues. Over recent years, our knowledge of the components of the RAS has increased, including numerous angiotensin peptides with diverse biological activities mediated by different receptor subtypes [1,2].

**Figure 1:**
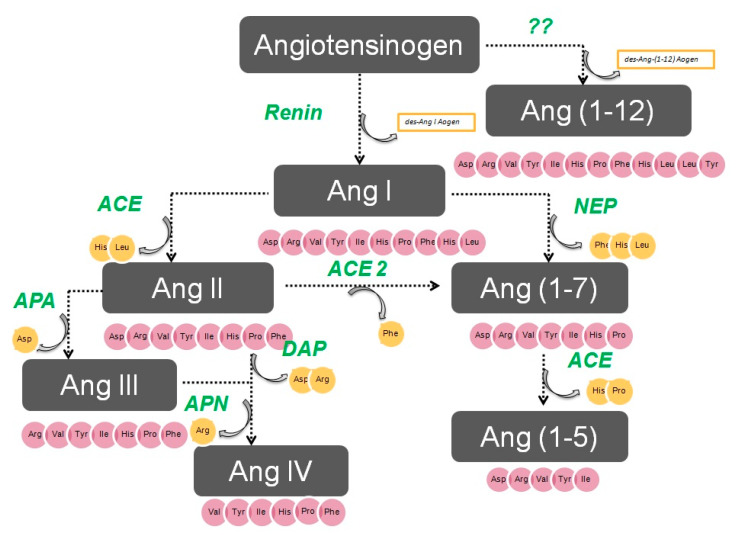
The renin–angiotensin system.

The enzymatic cascade of the RAS is depicted in Figure 1. It is initiated by angiotensinogen, an alfa 2 globulin of hepatic origin, which generates angiotensin I (AngI) through the enzymatic action of renin on the extreme amino-terminus. The decapeptide AngI is a substrate for angiotensin-converting enzyme (ACE), which splits the dipeptide His-Leu from the extreme carboxy-terminus to generate the octapeptide AngII [1], the major effector peptide of the RAS, which bind to two major receptors, AT1 and AT2, that generally oppose each other.

Action of glutamyl aminopeptidase (APA) on AngII removes the Asp residue N-terminus to generate the heptapeptide AngIII. AngIII can also be generated by an AngII-independent pathway via the nonapeptide [des-Asp1]AngI, produced from AngI by AspAP, which is converted to AngIII via ACE. APA also participates in the formation of Ang(1-7), which can also be transformed into Ang(2-7) through cleaving of the Asp-Arg bond.

In the extreme N-terminus, membrane alanyl aminopeptidase N (APN) removes Arg to give hexapeptide angiotensin IV (AngIV). AngIV is also transformed into Ang(3-7) by carboxypeptidase P (Carb-P) and propyl oligopeptidase through scission of the Pro-Phe carboxy-terminus.

ACE2 can also transform AngII into Ang(1-7) through hydrolysis of Phe by Carb-P or through scission of the dipeptide Phe-His from Ang(1-9) [1,2,3,4].

Aminopeptidase B (APB), also known as arginine aminopeptidase (Arg-AP), cleaves basic amino acids at the N-terminus. It participates in the conversion of AngIII to AngIV.

Although not part of the RAS, both neuropeptides oxytocin and vasopressin are cleaved at the N-terminus of cysteine next to tyrosine by cystinyl aminopeptidase (CAP), a rat homolog of insulin-regulated AP (IRAP) [5].

All these enzymes are globally called “angiotensinases’’ because they are responsible for the generation of systemic and local peptides (angiotensins) related to the regulation of BP and the excretion of sodium and water. These enzymes determine the proportions of bioactive compounds.

AngI is biologically inactive, but AngII and AngIII act as agonists for AT1 and AT2 receptors, thereby mediating pressor and dipsogenic effects [6,7]. AngIV has a low affinity for AT1 and AT2 receptors but high affinity and specificity for the AT4 receptor subtype. Interaction with the AT1 receptor subtype reduces the pressor effect of AngIV. A counterregulatory system to the AngII-AngIII/AT1 receptor system is composed of ACE2 and Ang(1-7), which activate the Mas receptor [6,7,8]. This system induces vasodilatory, antifibrotic, antihypertrophic, and antiproliferative effects.

## 2. Aminopeptidases in Arterial Hypertension

A list of the most common aminopeptidases with their enzyme commission (EC) numbers and their main abbreviations is shown in Table 1. As was introduced in the previous paragraph, aminopeptidases (Aps) generate the active compounds of the RAS and play an essential role in BP control and sodium handling [9]. APs can also degrade certain peptidergic hormones or neuropeptides such as vasopressin, cholecystokinin, and enkephalins [10]. For this reason, APs have been analyzed in plasma, kidney, and other tissues related to BP control in several rat models of hypertension.

Thus, reduced renal membrane-bound APA, iRAP, and APN activities were observed in a reduced renal mass saline model, and reduced APA activity was detected in a two-kidney one-clip Goldblatt hypertension model [11]. In the low renal mass model, a positive correlation was found in both soluble CAP and APN activities between the neurohypophysis and the adrenal gland, but this was not observed in the normotensive rats [12].

The relationship between APs and arterial hypertension has been explored with greater precision. For instance, it has been proposed that endoplasmic reticulum AP 1 (ERAP1) and ERAP2 regulate BP by inactivation of AngII, and these two APs, which also hydrolyze amino acids from the N-terminus of various human antigens and peptide hormones, are widely expressed in human tissues, including heart, endothelial cells, and kidney [13,14,15]. In vitro, ERAP1 transforms AngII into AngIII and AngIV [14], while ERAP2 converts AngIII to AngIV [15]. ERAP 1 was initially identified as a placental leucine AP that degrades AngII and III and transforms kallidin into bradykinin in vitro, thus has a role in BP regulation [14].

In this sense, several interesting papers have been published. In an in vivo study, an increase in circulating levels of ERAP1 was found to reduce BP and AngII levels [16]. In a genetic study, an association was detected between variants of the gene encoding Arg528 and the development of essential hypertension in a Japanese population [17]. In an investigation of 45 genetic variants of ERAP1 and ERAP2 in 17,255 Caucasian females from the Women’s Genome Health Study, ERAP1 was found to be related to increased BP [18]. The ERAP1 genotype also appears to be involved in the reduction of left ventricular mass produced by some antihypertensive treatments [19]. Thus, all these data indicate a possible role for ERAP1 in BP regulation and ventricular remodeling. Finally, other genetic studies have associated variants of ERAP1 and ERAP2 genes with preeclampsia [20], hemolytic uremia [21], and hypertension [17].

### Aminopeptidases after Antihypertensive Therapy

Various systemic antihypertensive treatments can alter the activities of brain and systemic APs associated with effects on BP. Thus, in a rat study by Banegas et al. [22], unilateral brain lesions in the nigrostriatal system produced simultaneous and paralleled changes in BP and in brain and plasma AP activities. Later, the same group [23] examined the participation of APs in the metabolism of some angiotensins, vasopressin, cholecystokinin, and enkephalins in the plasma and hypothalamus of spontaneous hypertensive (SHR) rats under normal conditions and after beta-blocker treatment with propranolol. In this rat strain, AP activity in response to propranolol administration differed between plasma and hypothalamus, thus suggesting an interaction between APs and the autonomic nervous system. Moreover, these authors also reported that treatment with ACE inhibitors captopril, propranolol, or the nitric oxide synthesis inhibitor L-NAME, induced a marked modification of brain patterns of neuropeptidase activity in SHR rats [24].

The BP-lowering effect of a diet enriched in extra virgin olive oil in rats was analyzed by Villarejo et al. [25] who observed that rats receiving this diet showed augmented APN and AspAP activity in the renal cortex, suggesting a greater degradation of AngIII and AngIV and an increased generation of the antihypertensive Ang(2-10). Hence, the glomerular formation of Ang(2-10) might compensate the well-known pressor effects of AngII on the glomerular vasculature in this model of hypertension.

Taken together, the data reported in this section indicate that BP changes induced by antihypertensive or prohypertensive drugs are associated with modifications in AP activity. However, no definitive conclusions can be drawn about the functional role of APs in the pathogenesis of hypertension.

## 3. Brain APA

Aminopeptidase A (APA) is a 109 kDa homodimeric zinc-metallopeptidase that catalyzes the cleavage of glutamatic and aspartatic amino acid residues from the N-terminus of polypeptides. It is encoded by the ENPEP gene and is also known as glutamyl aminopeptidase, gp160, or CD249.

**Figure 2:**
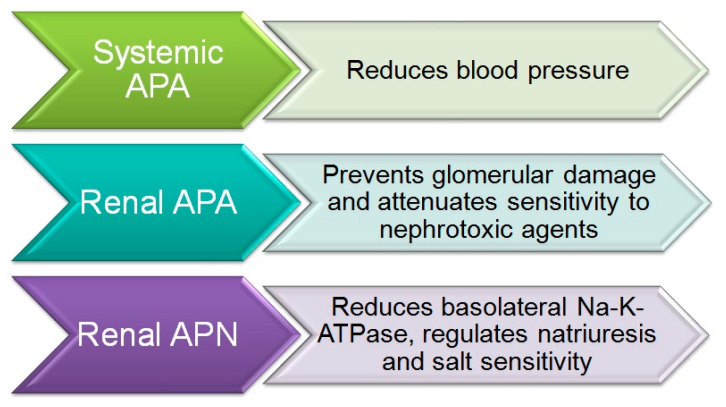
Systemic aminopeptidase A (APA) and aminopeptidase N (APN) in blood pressure control.

Numerous studies have demonstrated the major role of brain AngIII and both APA and APN in the control of BP and in arterial hypertension [26]. Thus, the intracerebroventricular (icv) injection of APA to induce the transformation of AngII into AngIII was found to elevate BP in normotensive WKY and SHR animals [27]. In contrast, the icv administration of APN, which hydrolyzes AngIII, reduced the BP in WKY rats and to a greater degree in SHR animals [28]. A study of the icv administration of analogs of AngII and AngIII to SHR rats found that a greater BP increase was induced by AngIII than with AngII [29], thus indicating that a major BP reduction is produced by the inhibition of APA activity and consequent interference in the transformation of AngII to AngIII [30], which suggests a greater contribution of brain AngIII than AngII to the BP increase (Figure 2).

### Systemic and Renal APA

APA is a membrane-bound enzyme with a major presence in the kidney, in multiple tissues [31], and in a soluble form in the blood, probably due to the cleavage of membrane-bound APA [32]. APA, which has also been called angiotensinase [33], transforms AngII, the most active systemic peptide, to AngIII, limiting the rate of angiotensin generation [34,35].

The importance of APA in BP control is evidenced by the BP reduction that follows the administration of purified APA [1] and by the BP increase induced by APA inhibitors [36]. In addition, APA-deficient mice are characterized by a high BP and an increased responsiveness to AngII [37]. These data support a role for APA in the regulation of BP under physiological conditions and in hypertensive humans and experimental models.

A study of SHR rats found that a decrease in kidney APA was related to an increase in RAS activity, whereas the administration of APA produced a dose-related reduction in systolic BP [38], showing a 2300-fold increase in activity in comparison to the AT1 blocker candesartan [39]. APA abnormalities have also been observed in the Goldblatt hypertension model [40]. Thus, Prieto et al. [41] reported decreased APA levels in the renal cortex of clipped and non-clipped kidneys in this model, suggesting the involvement of APA in augmenting the AngII-induced reabsorption of sodium and water. Renal RAS is also increased in Dahl salt-sensitive (DSS) rats [42]; in this model, age-related glomerular injury is associated with an increasing elevation of AngII levels because sclerotic glomeruli are less active in synthesizing APA [43]. In human subjects, serum APA activity increases in an age-dependent manner in both men and women [44], and this may be in relation to the metabolic clearance of AngII [45].

At the renal level, Velez et al. [46] observed an increased sensitivity to glomerular damage in APA-deficient BALB/c mice. The authors injected the APA-knockout (KO) mice with a nephrotoxic serum and observed glomerular hyalinosis and albuminuria at 96 h post-administration, whereas no renal injury was observed in the wild-type controls. Likewise, the 4-week infusion of AngII reduced podocyte nephrin levels in APA-KO mice but not in wild-type controls. These data indicate that the degradation of AngII induced by APA plays a protective role in glomerular injury.

Taken together, the above data indicate that an increase in systemic APA protects against hypertension. Conversely, a reduction in the activity of this enzyme maintains high levels of AngII and therefore promotes hypertension (Figure 2). As a conclusion, increased APA activity in the brain raises BP through an increased generation of AngIII, which is the main AT1 receptor agonist in the brain, whereas increased APA activity in the peripheral circulation lowers BP through the degradation of AngII (Figure 2).

## 4. APN

Aminopeptidase N (APN), also called leucine aminopeptidase and alanyl aminopeptidase, is a homodimeric, membrane-bound, zinc-dependent aminopeptidase [47]. APN cleaves AngIII to AngIV via the scission of arginine at the extreme N-terminus, which indicates its participation in the regulation of tissue and systemic RAS [48]. APN is highly expressed in the central nervous system and kidneys [47,49,50] and may develop multiple actions [47] besides peptide cleavage [51,52]. For this reason, APN is known as a “moonlighting protein” [53].

### 4.1. Brain APN in BP Regulation

**Figure 3:**
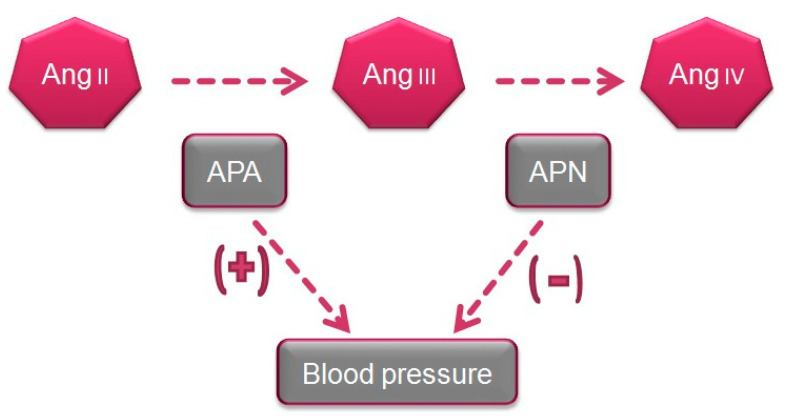
Systemic and renal APA and APN in the control of blood pressure and renal function.

Studies in rodents suggest the participation of brain APN in BP regulation [54]. Thus, icv administration of APN decreased BP in WKY and SHR animals, with more effect in the hypersensitive rats [55]. Conversely, icv administration of bestatin and amistatin, APN inhibitors, raised BP and induced a dipsogenic response in WKY and SHR animals (Figure 3) [55]. The paraventricular nucleus of the hypothalamus appears to be the target for APN in the brain, because its microinfusion at this site reduced BP in both SHR and WKY rats [56,57]. Central APN exerts its effects by transforming the pressor AngIII in the brain into AngIV. In this way, the pressor response induced by icv AngIII is potentiated by the APN antagonists bestatin and amistatin [38,58]. In line with these observations, administration of an angiotensin antagonist was found to inhibit the increase in BP produced by the icv administration of APN inhibitors [30,57].

### 4.2. Renal APN in BP Control

APN is widely distributed in the kidney and has been detected in glomeruli, mesangial cells, and on the luminal surface of tubules [47,58]. Renal APN generates AngIV [59,60], and infusion of AngIV in the renal artery increased sodium excretion [61,62,63], an effect related to the reduced activity of basolateral tubular Na-K-ATPase [64]. This mechanism may also participate in the adaptative response to increased salt intake, thus protecting against hypertension. Moreover, abnormalities in renal APN have also been observed in several models of experimental hypertension. Thus, the Goldblatt model showed increased APN activity in the renal cortex of the non-clipped kidney [41] and APN protein abundance and activity in the kidney were increased in Dahl salt-resistant versus Dahl salt-sensitive animals (64). APN has also been associated with essential hypertension in humans [65].

#### APN as a Regulator of Salt Sensitivity

Renal APN regulates mechanisms that facilitate renal sodium excretion after increased saline intake, producing a coordinated decrease in Na-K-ATPase abundance on the basal side of the tubule (by endocytosis) and a reduction in sodium transporters (by internalization) [66,67,68].

APN abundance is higher in Dahl salt-resistant rats. In these animals, APN may reduce basolateral Na^+^-K^+^-ATPase as a protective mechanism in response to the increased saline intake [64]. Reduced tissue and plasma APN levels have also been reported in the L-NAME model but not in the controls [68]. In conclusion, renal tubule levels of APN can regulate sodium excretion and, therefore, salt sensitivity and BP.

### 4.3. Other Actions of APN Related to the Cardiovascular System

APN activation has been reported in diabetic nephropathy, renal damage, connective vascular disease, and cerebral ischemia [69]. In mice, APN is also essential for inflammatory trafficking after coronary artery occlusion and for sustaining the reparative response [70]. Hence, APN blockade of APN is a therapeutic approach to these vascular abnormalities.

Stimulation of AT4 receptors by APN-generated AngIV exerts proangiogenic action. Thus, APN is augmented in pathologic angiogenesis, especially in tumor vasculature [71], and APN blockade reduces angiogenesis in vivo [72]. In APN-null mice, angiogenesis alterations are manifested in pathological situations but not under physiological conditions [73]. According to these observations, APN activity promotes angiogenesis in various conditions and its blockade prevents new blood vessel growth. Indeed, molecular imaging of APN has been used to detect and monitor multiple types of cancer and the surface of vasculature undergoing angiogenesis in cardiac regeneration [74]. Hence, APN is a potential biomarker of angiogenesis and therapeutic tool.

## 5. Therapeutic Strategies to Treat Arterial Hypertension with Aminopeptidases

The vast majority of studies on the control of BP and treatment of hypertension have addressed the blockade of AngII or its receptors, and there has been less research on the regulation of other angiotensin peptides. Thus, blockade of the brain RAS has been found to simultaneously decrease sympathetic tone, vasopressin release, and baroreflex activity, thereby reducing cardiac output and peripheral resistance [75].

An action on central or peripheral APs represents a new approach to the treatment of hypertension. Thus, new antihypertensive treatments have been developed based on potent orally-active inhibitors of APA or activators of aspartyl-aminopeptidase (DNPEP), since brain aspartyl aminopeptidase exerts BP-lowering effects by transforming AngI into angiotensin 2-10. Currently, the search for new antihypertensive compounds that affect the RAS multi-enzyme cascade is an important line of research.

### 5.1. Inhibition of APA

The icv administration of EC33, a specific APA inhibitor, prevented the BP increase produced by the icv administration of AngII in SHR animals, indicating that the central response to AngII requires its transformation into AngIII by APA. A marked BP decrease has also been observed in conscious SHR and DOCA-salt hypertensive rats after the icv infusion of EC33 [76,77]. In contrast, the peripheral iv infusion of EC33 did not reduce BP, indicating that EC33 does not cross the blood–brain barrier and/or is inactive in systemic circulation.

The BP of SHR rats increased after the central icv administration of APA [78], probably due to an increased endogenous generation of AngIII, whereas APA blockade with an antiserum attenuated the pressor response to AngII by around 60% [78]. It is interesting to note that a selective APA inhibitor (RB150) with antihypertensive properties can be given either intravenously [77] or orally [79] because it can cross the blood–brain barrier.

The peripheral activity of the AT1 receptor depends on the transformation of AngII into AngIII by APA [80]. Thus, antihypertensive effects were observed in SHR rats after the systemic administration of recombinant APA [81] at a dose that was one-tenth of the usual candesartan dose [82]; the joint i.v. administration of APA and APN attenuated the pressor effect of AngII in normal rats and treatment with APA reduced the BP of SHR rats to normal levels [83].

Considered together, these data clearly demonstrate that APA reduces BP, while abnormalities in APA activity promote hypertension, as supported by the lower renal APA activity in SHR versus WKY rats [84]. The administration of APA has therefore been proposed for the treatment of acute heart failure, acute hypertensive crisis, preeclampsia, and hypertensive encephalopathy, among other hypertensive emergencies [85,86].

### 5.2. APN Blockade in the Treatment of Hypertension

The administration of PC18, an inhibitor of APN, generates a pressor response through the accumulation of endogenous AngIII, which is mediated via the AT1 receptor. In this way, pretreatment with the AT1 blocker losartan can suppress the pressor response, while the AT2 antagonist PD123319 is unable to prevent the BP increase [87]. The enhanced proximal tubular sodium reabsorption of SHR rats is prevented by the intrarenal infusion of PC18 [88]. This finding indicates that the blockade of AngIII degradation achieved by APN inhibition improved sodium excretion in the proximal tubule of these rats when it was administered in the renal interstitium [89]. Hence, the transformation of AngII into AngIII is required for this natriuretic response, which is not affected by the AT1 blocker candesartan. Research on the usefulness of APN inhibitors to treat hypertensive patients is at an early stage, and further studies are required.

## 6. APs as Urinary Biomarkers of Renal Injury

Serum creatinine and blood urea nitrogen (BUN) are widely used markers of renal disease, but their sensitivity and specificity are limited, and they are not useful in distinguishing the stages of acute kidney injury (AKI) [90]. They lack sensitivity because they increase only when the renal lesion is evident. Thus, serum creatinine levels rise gradually, and the kidneys have already lost half of their functionality by the time normal levels are doubled [91,92]. Besides, normal levels of these markers can be affected by protein-rich diets, intestinal bleeding, muscle disease, and dehydration, generating false positives in the diagnosis of renal disease. There is a need for biomarkers to allow an earlier diagnosis of AKI, a better prediction of renal disease severity, and an improved assessment of adverse effects in drug development [90].

Various urinary biomarkers for the early detection of AKI have emerged over recent years [91,92], including tubular enzymes that are increased in urine after damage to the tubular epithelium [92], which can precede or even trigger renal dysfunction. Major advantages of urinary markers include the non-invasiveness of the sampling and their usefulness to elucidate the size and localization of tubular cell lesions and to detect the presence of necrosis or other alterations that evoke renal dysfunction [93]. The measurement of urinary enzymes and other urinary biomarkers may therefore be a valuable tool to obtain an early diagnosis during initial stages of renal disease and to follow its progression or regression, facilitating prediction of the prognosis. Urinary APs are considered promising and useful biomarkers of renal disease of different pathophysiological origin, and an automated photometric assay has been developed for their measurement [94].

**Figure 4:**
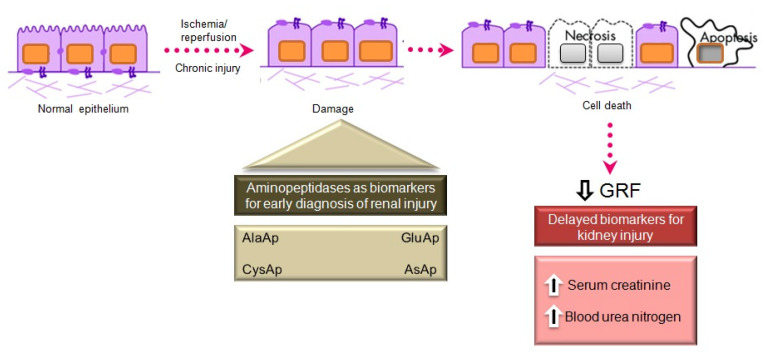
Main aminopeptidases studied as biomarkers of acute and chronic kidney injury.

APA, APN, and CAP are present in the brush border membrane of renal tubular cells [95], have molecular weights above 140 kDa, and are highly organ-specific. These conditions ensure the tubular origin of these urinary enzymes, which cannot pass easily into the urine through the glomerular barrier (Figure 4).

APs participate in AngII metabolism, forming part of the renal renin–angiotensin–aldosterone system (RAAS) [96], which is elevated in renal diseases. One of these enzymes, alanine aminopeptidase (APN), is a brush border enzyme that was proposed in the early 1970s as a urinary marker of renal disease [97]. Later, Marchewka et al. [98] demonstrated that the measurement of APN and its isoforms was of diagnostic relevance in nephrolithiasis. They also observed significantly higher APN excretion in patients with glomerulonephritis than in controls and a positive correlation between urinary protein concentrations and APN activity [99]. Their findings supported the association between proteinuria and elevated activity of renal tubular brush border enzymes reported in other studies of chronic glomerulonephritis [100]. As an explanation of this association, these authors [99] proposed that the protein present in the ultrafiltrate produces a release of APN from the external membrane of renal tubule microvilli. Because of its external localization, its release into the urine can be caused by a weak destructive action and is not necessarily linked to disruption of the integrity of kidney tubule cells [101].

Jung et al. [102] reported that the urinary excretion of APN, alkaline phosphatase, γ-glutamyltransferase, and N-acetyl-β-D-glucosaminidase is age-dependent in humans. These enzymes were determined in random morning urine samples from 442 individuals aged from 5 days to 58 years, and their creatinine-normalized activity significantly decreased with increasing age. APN activity has also been proposed as a biomarker of the nephrotoxicity induced by vancomycin [101] or amphotericin B [103] in experimental models and in several human diseases, such as glomerulopathy [104], IgA nephropathy [105], and diabetes [106]. However, conflicting results have been published on its usefulness as a biomarker of renal function in kidney transplantation patients [107,108].

### Acute Kidney Injury

AKI is defined by an abrupt increase in serum creatinine during the 48 h period after an insult responsible for functional or structural changes in the kidney, and it is mainly caused by the acute apoptosis or necrosis of renal tubular cells [109]. The early detection of AKI is a current preclinical and clinical research priority. The administration of nephrotoxic drugs that alter tubular function is the most common experimental model to relate the excretion of different biomarkers to renal dysfunction. Cisplatin is an antineoplastic drug with a potent nephrotoxic effect, mainly due to its action on the proximal tubule, causing AKI in experimental animal models and patients [110,111]. Its administration induces tubular and glomerular disturbances that lead to the development of interstitial fibrosis [112].

**Figure 5:**
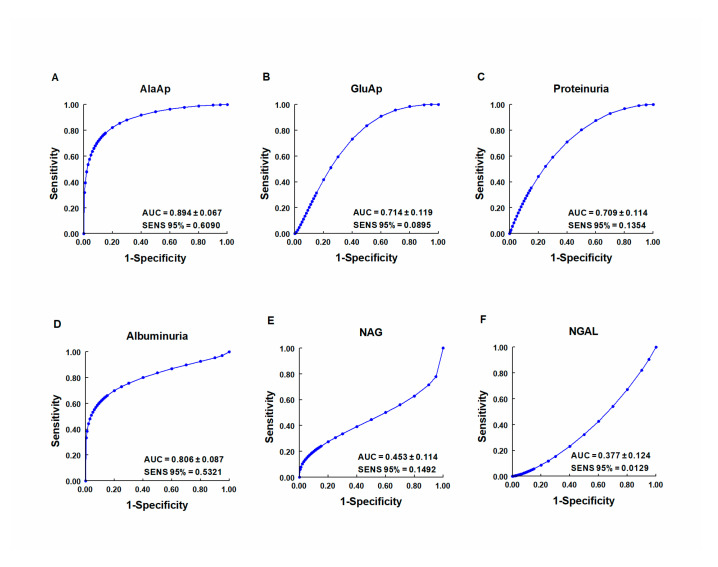
ROC curves showing specificity and sensitivity for APN (AlaAp) (**A**), APA (GluAp) (**B**), proteinuria (**C**), albuminuria (**D**), N-acetyl-β-D-glucosaminidase (NAG) (**E**), and neutrophil gelatinase-associated lipocalin (NGAL) (**F**) to differentiate cisplatin-treated rats from control rats 24 h after injection of saline, 3.5 or 7 mg/kg of cisplatin (*n* = 8 each group). All cisplatin-treated rats displayed tubular dysplasia and interstitial fibrosis 14 days after injection. Urinary markers were expressed in daily total activity or excretion per 100 g of body weight. AUC = area under the curve. SENS 95 % = calculated sensitivity at 95 % of specificity.

Our group investigated whether urinary activities of APN, APA, and CAP could serve as biomarkers of renal dysfunction by using a rat model of cisplatin-induced nephrotoxicity [113]. Their activity was determined in urine samples collected at 24, 48, and 72 h after cisplatin injection. Their urinary activity at 24 h post-injection was correlated with two renal function indicators (plasma creatinine and creatinine clearance) and their activity at two weeks post-injection was correlated with two indicators of structural damage (renal hypertrophy and interstitial fibrosis). A comparative analysis was also performed with other proposed urinary biomarkers of kidney injury, i.e., albumin, proteinuria, NAG, and NGAL. The area under the curve (AUC) was calculated to quantify the sensitivity and specificity of each marker to distinguish cisplatin-treated from control rats at 24 h post-injection (Figure 5). APN, APA, CAP, and DNPEP had an AUC value greater than 0.5, indicating that these enzymes are early biomarkers of kidney injury in cisplatin-treated rats. The greatest specificity and sensitivity values were observed for APN, which was the best variable to discriminate between treated and untreated animals, whereas an AUC value below 0.5 was observed for NAG and NGAL at this time point. A significant correlation was also found between AP activities at day 1 post-injection and functional and structural variables at day 14. It can therefore be concluded that the urinary activities of these APs may serve as early and predictive biomarkers of cisplatin-induced renal dysfunction. The measurement of urinary AP activities can be useful in preclinical research for the early detection and follow-up of renal damage and for the evaluation of drug nephrotoxicity, and it may offer a useful prognostic and diagnostic tool for renal diseases in the clinical setting.

## 7. Quantification of Urinary APs

APs activities can be readily quantified in biological samples by kinetic methods using photometric [94] or fluorometric [113] assays. Widely applied immunological techniques such as immunoblotting or ELISA can be used to analyze the amount/expression of these proteins in biological samples. Our group used ELISA and Western blot to measure the amount of APN excreted in the urine of cisplatin-treated rats and correlated its excretion 24 h post-injection with increased serum creatinine and decreased body weight at two weeks [114]. In addition, the quantification of APN by ELISA showed a very high correlation with urinary APA activity. The above findings confirm that the immunological determination of this enzyme can also serve as an early marker of renal damage. Further studies are warranted to compare the sensitivity and specificity of kinetic and fluorometric methods in other experimental models and in human diseases that involve renal dysfunction.

### 7.1. Aminopeptidases in Urinary Vesicles and Exosomes

Extracellular vesicles (EVs) released by the renal epithelium into the tubular fluid are excreted and may be detected in urine samples [115,116]; they include microvesicles and exosomes. Exosomes are nanovesicles (30–120 nm) released by epithelial cells throughout the urinary tract [117,118]. Thus, the exosomic fraction of urine contains potential biomarkers of various renal diseases. Since exosomes secrete mRNAs, miRNAs, and proteins that affect the function of recipient cells [119], it has been proposed that the biological role of exosomes in the kidney is to regulate the co-functionality of different sections of the nephron.

However, the content of biomarkers in urinary microvesicles has not been fully elucidated. The release of microvesicles into the urine has been studied in some renal diseases [120], and their content can also transmit biological signals within the kidney [121,122,123,124].

**Figure 6:**
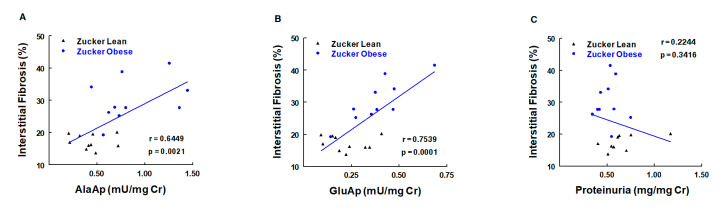
Correlations of urinary activities of APN (AlaAp) (**A**), APA (GluAp) (**B**), and proteinuria (**C**) with percentage of renal interstitial fibrosis in urine samples of Zucker lean and obese rats (*n* = 10 each group). Cr = creatinine (adapted from reference [126]).

Our group therefore investigated whether the microvesicular and exosomic content of APA in urine was related to cisplatin-induced renal dysfunction in rats [125]. An increased APA content was observed in microvesicular and exosomic fractions at the peak of toxicity, 3 days post-cisplatin injection, and it showed a significant predictive correlation with serum creatinine, regardless of the method of normalization used for APA values (Figure 6). It was concluded that APA content and activity in urinary microvesicles and exosomes may serve as renal dysfunction biomarkers in this nephrotoxicity model.

Thus, the determination of GluAp in microvesicular and exosomic fractions can be useful to study proximal tubular lesions independently of the glomerular filtration rate. From a technical viewpoint, it is interesting to note that the microvesicular fraction can be obtained more rapidly than can the exosomic fraction. In addition, the analysis of these fractions can avoid interference due to urea content, ionic strength, or other components of urine, because the samples are centrifuged and dissolved in a buffer. According to these observations, examination of the content of the microvesicular fraction can also be a relevant diagnostic tool in nephropathies.

### 7.2. AKI Induced by Surgical Procedures

Patients undergoing a cardiopulmonary bypass (CPB) can develop AKI as a post-surgical complication. The diagnosis of AKI in these patients is based on an increase in serum creatinine (SCr) concentration over baseline values during the 48 h postoperative period; however, the slow progressive rise of SCr can delay the detection of renal dysfunction. Kim et al. [127] analyzed the serum and urinary enzymatic activity of vasopressinase and SCr in 31 patients at different time points after CPB surgery. Results showed maximum serum and urine activity of vasopressinase at intensive care unit (ICU) admission in patients developing AKI as a complication of CPB earlier than the peak of SCr activity, which is usually observed at 48 h post-surgery. The authors therefore proposed vasopressinase (cystil AP, CAP) activity as an early biomarker for AKI. Animal models of renal ischemia also showed that AKI was related to vasopressinase activity [128].

In a similar study of 103 patients, our group observed a significant increase in urinary APA activity at ICU admission in patients who developed persistent AKI in comparison to those who did not experience AKI or developed only transient AKI. Urinary APA activity showed a higher sensitivity than SCr at this time point, obtaining a higher total number of correctly diagnosed cases in comparison to the application of criteria published by the Acute Kidney Injury Network [129]. These observations also indicate that tubular injury increases the risk of post-surgery AKI.

### 7.3. Urinary Aminopeptidases as Biomarkers of Renal Dysfunction in Chronic Diseases

#### 7.3.1. Obesity

Obesity and hypertension are chronic diseases that can ultimately lead to renal failure. Experimental models of these diseases have been used for long-term evaluation of the usefulness of urinary APs as renal injury biomarkers.

The obese Zucker rat is an animal model of human type II diabetes [130] characterized by obesity, dyslipidemia, insulin resistance, and kidney damage. Male obese (ZO) and lean (ZL) Zucker rats were studied for 6 months (age of 2–8 months) [131]. At the age of 8 months, ZO rats exhibited various renal lesions, including mild focal and segmental glomerulosclerosis and moderate tubulointerstitial damage. Urinary GluAp (APA) and AlaAp (APN) activities were measured monthly and were increased in ZO rats. Both APA and APN activities correlated with renal lesions and can be considered early diagnostic biomarkers of these lesions (Figure 6). AP activities also showed predictive correlations with the level of interstitial fibrosis in the kidney and with the amount of hydroxyproline accumulated in renal tissue at the age of 8 months. Hence, the early excretion of these markers also serves to evaluate the development of fibrosis in this experimental model.

#### 7.3.2. Hypertension

The SHR model of essential hypertension in humans is widely used [132,133] and is characterized by an increase in BP and development of renal injury as age advances [126,133]. This model has been used to analyze the urinary enzymatic activities of APA, APN, and dipeptidyl peptidase-4 (DPP4) as biomarkers of renal injury in hypertension. These activities were measured in male SHR and WKY rats from the age of 2 to 8 months [134]. The SHR animals did not develop relevant signs of histopathological kidney alterations at the end of the study, but they showed increased glomerular area and cellularity. These activities were increased in monthly-collected urine samples from SHR rats and were correlated with systolic blood pressure throughout the study. Their increased activity in SHR animals indicate that a certain degree of tubular injury can be detected before the appearance of all histopathological manifestations of renal disease. These markers can therefore be used for the diagnosis of renal disease in early stages of hypertension and offer a non-invasive method to follow the progression of renal dysfunction in hypertension and other diseases.

#### 7.3.3. Hyperthyroidism

In rats, hyperthyroidism is an endocrine disease associated with hypertension and renal abnormalities, accelerating the course of experimental hypertension, whereas hypothyroidism is associated with hypotension, preventing the development of hypertension and protecting against renal injury [135]. Ethanol-binding protein and N-acetyl-β-D-glycosaminidase, urinary biomarkers of tubular damage, are increased in hyperthyroid humans [136] and cats [137].

Our group studied the urinary excretion of APN, APA, CAP, and AspAp in control, hyperthyroid, and hypothyroid rats receiving a normal- or high-sodium diet. All APs were augmented in hyperthyroid rats, whereas their levels were similar between hypothyroid and control rats [138]. Hyperthyroid rats receiving a high sodium diet showed increased hypertension, cardiac/renal hypertrophy, albuminuria, and oxidative stress and a marked rise in urinary AP activities. AP activity was modestly elevated in the controls on a high-sodium diet but did not differ between hypothyroid rats on a high- versus normal-sodium diet. According to these results, the combination of T4 treatment and salt exert additive effects on the production of tubular damage, whereas hypothyroidism confers resistance to the effects of high salt intake on urinary AP activities. These findings support previous descriptions of hypothyroid status as beneficial in chronic kidney diseases [139]. It can be concluded that urinary AP activities are diagnostic biomarkers of renal injury in this experimental model. Our group [140] also determined APA activities in renal tissue and plasma samples from adult male euthyroid, hyperthyroid, and hypothyroid rats, and found a significantly higher expression of renal APA in hyperthyroid versus control or hypothyroid rats. However, plasma APA activity was lower in hyperthyroid versus control rats, indicating that the increased APA in these animals is focalized in renal tissue.

#### 7.3.4. Diabetes Mellitus

In a study of urinary levels of enzymes and low-molecular-mass proteins as indicators of diabetes nephropathy, Jung et al. [141] observed an increase in urinary alanine aminopeptidase in diabetic patients and an even greater increase in diabetic patients with proteinuria. However, a study by Lazarevic et al. [106] on the impact of aerobic exercise on microalbuminuria and enzymuria in type II diabetic patients found no significant difference in urinary or plasma APN activities over the study period between patients with and without diabetes or within the diabetes group. Numerous factors may be implicated in these discrepancies between studies.

#### 7.3.5. Urinary APs in Other Chronic Diseases

Sorokman et al. [142] measured urinary levels of renal-specific enzymes (neutral α-glucosidase, L-alanine aminopeptidase, and γ-glutamyltranspeptidase) as markers of proximal tubule damage in children with pyelonephritis, the most common cause of fever in patients with urinary system disorders. They observed an increase in urinary levels of these enzymes during the active phase of pyelonephritis, which correlated with leukocyturia and C-Reactive Protein levels. Finally, Spasovski et al. [143] studied urinary levels of brush border enzymes of the proximal renal tubules (e.g., APN, gamma-glutamyl transferase, and beta2 microglobulin) in patients with untreated rheumatoid arthritis to explore the possible relationship between this disease and dysfunction of the brush border of proximal tubules. They found that urinary APN activity was a superior indicator of asymptomatic renal lesions in untreated rheumatoid arthritis patients compared with measurements of gamma-GT or beta2m.

## Figures and Tables

**Table 1:** Types of aminopeptidases showing their most common abbreviations.

Enzyme	EC Number	Abbreviations
Leucyl AP	3.4.11.1	LAP
Membrane alanyl AP	3.4.11.2	APN, AlaAP
Cystinyl AP	3.4.11.3	CysAP, CAP
Prolyl AP	3.4.11.5	PIP
Aminopeptidase B	3.4.11.6	APB, ArgAP
Glutamyl AP	3.4.11.7	APA, GluAP, EAP
Aminopeptidase P	3.4.11.9	APP
Cytosol alanyl AP	3.4.11.14	AAP, AlaAP
Methionyl AP	3.4.11.18	eMetAP
Aspartyl AP	3.4.11.21	AspAP, DNPEP
Arginyl AP	3.4.22.16	iRAP, APR

## Abbreviations

ACE	Angiotensin converting enzyme
AKI	Acute kidney injury
AngI	Angiotensin I
AngII	Angiotensin II
AngIII	Angiotensin III
AngIV	Angiotensin IV
APs	Aminopeptidases
APA	Aminopeptidase A
APN	Aminopeptidase N
AT1	Angiotensin receptor type 1
AT2	Angiotensin receptor type 2
BP	Blood pressure
CAP	Cystinil AP
DOCA	Deoxycorticosterone
L-NAME	N(ω)-nitro-L-arginine methyl ester
RAS	Renin–angiotensin system
SHR	Spontaneous hypertensive rat
WKY	Wistar Kyoto rat
ZO	Zucker obese rats
